# Disappearance of multiple pancreatic cysts after prednisolone treatment in a patient with autoimmune pancreatitis

**DOI:** 10.1002/ccr3.1737

**Published:** 2018-07-22

**Authors:** Junji Kohisa, Atsunori Tsuchiya, Masatoshi Ikemi, Shuji Terai

**Affiliations:** ^1^ Division of Gastroenterology and Hepatology Graduate School of Medical and Dental Science Niigata University Niigata Japan; ^2^ Division of Gastroenterology and Hepatology Sado General Hospital Niigata Japan

**Keywords:** autoimmune pancreatitis, chronic pancreatitis, pancreatic cysts, prednisolone

## Abstract

Autoimmune pancreatitis (AIP) with multiple pancreatic cysts is rare. The severe narrowing of the branched pancreatic ducts found in active AIP with a chronic pancreatitis background may have caused pancreatic juice outflow obstruction, resulting in multiple cysts. Oral steroid therapy resolved the stenosis, resulting in disappearance of the cysts.

A 76‐year‐old man with a history of chronic pancreatitis due to alcohol abuse was referred to our hospital for epigastric discomfort. Abnormal laboratory variables included serum amylase, 1002 IU/L; lipase, 1533 mg/dL; IgG, 3878 mg/dL; IgG4, 1410 mg/dL; and a high titer of antinuclear antibody (×1280). Abdominal ultrasonography, enhanced computed tomography, and magnetic resonance cholangiopancreatography (MRCP) showed diffuse pancreatic swelling, 13 pancreatic cysts and calcification (Figures 1, 2 and 3). Endoscopic retrograde pancreatography showed irregular narrowing and disruption of the pancreatic duct (Figure 4). We could not obtain fluid from the cysts. He was diagnosed as autoimmune pancreatitis (AIP) with multiple pancreatic cysts. Treatment was initiated with 35 mg/d of prednisolone and gradually tapered down by 5 mg. One year later, MRCP revealed that the pancreatic cysts had almost disappeared with concomitant resolution of the pancreatic swelling and stenosis of the pancreatic duct (Figure 5).

**Figure 1:**
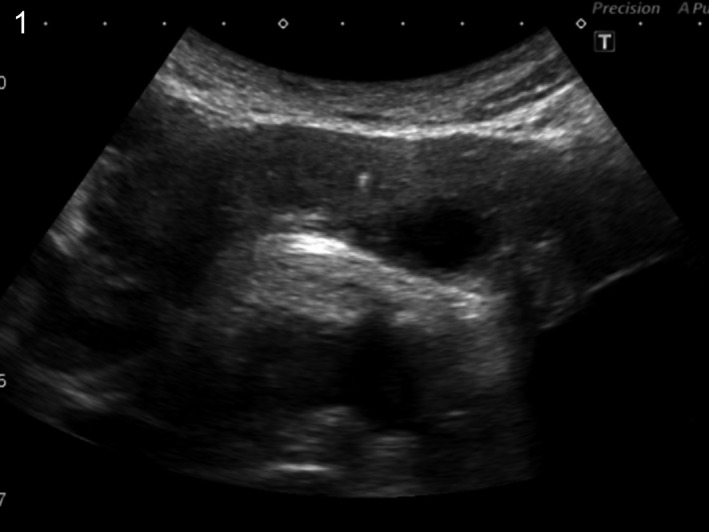
Abdominal ultrasonography showed a spot of high echogenicity in the pancreas indicating calcification secondary to chronic pancreatitis

**Figure 2:**
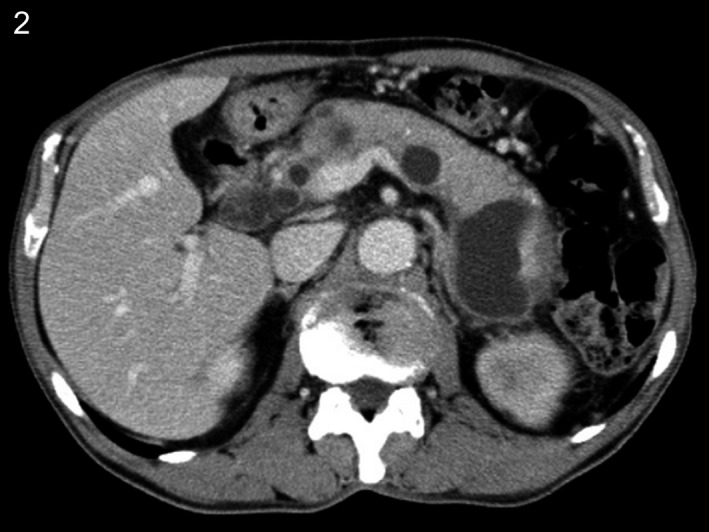
Computed tomography showed diffuse swelling of the pancreas, multiple cysts from the head to the tail, and calcification

**Figure 3:**
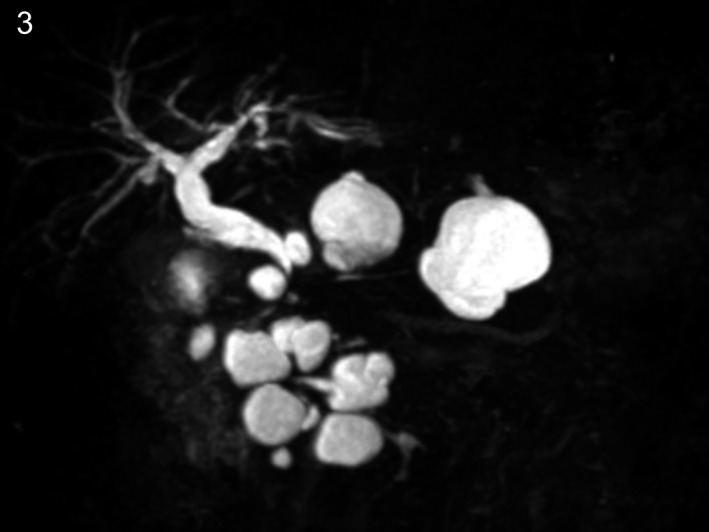
Magnetic resonance cholangiopancreatography revealed more than 10 pancreatic cysts with a maximum diameter of 42 mm

**Figure 4:**
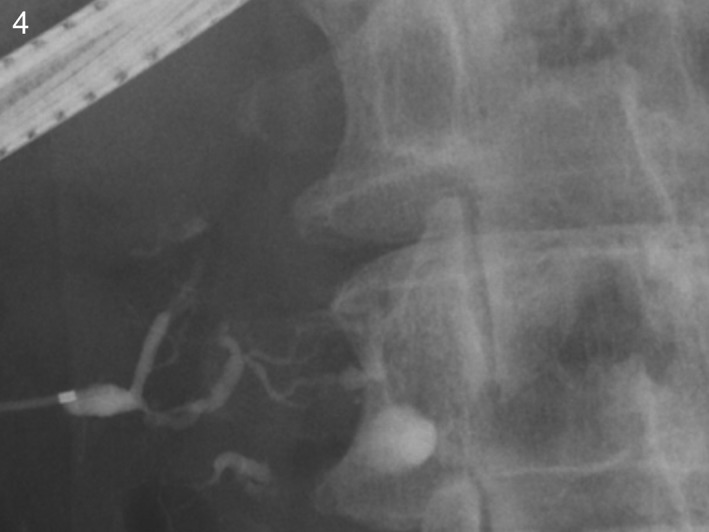
Endoscopic retrograde pancreatography showed irregular narrowing and disruption of the pancreatic duct

**Figure 5:**
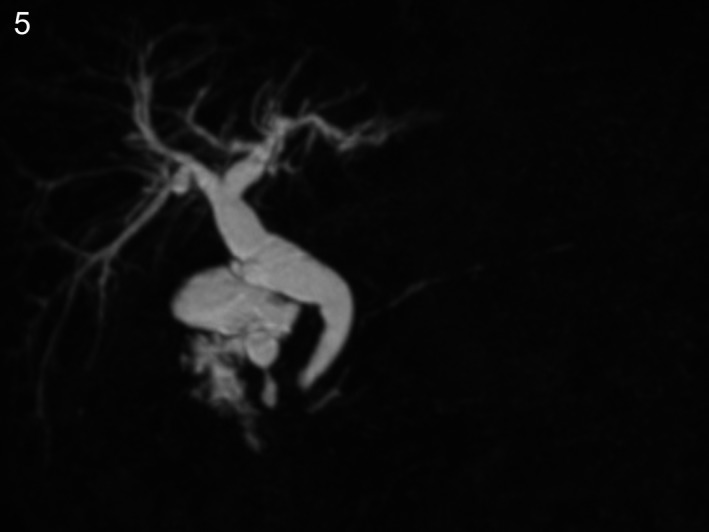
Magnetic resonance cholangiopancreatography after prednisolone treatment showing almost complete disappearance of multiple pancreatic cysts

There have been recent reports of AIP with a few pancreatic cysts; however, AIP with multiple pancreatic cysts are rare.^1^, ^2^ In this case, we concluded that AIP occurring in a setting of chronic pancreatitis (rather than isolated AIP or isolated alcoholic chronic pancreatitis) caused the multiple cysts. The severe narrowing of the branched pancreatic ducts found in active AIP with a chronic pancreatitis background may have caused pancreatic juice outflow obstruction, as indicated by the elevation of serum levels of amylase and lipase, resulting in the multiple pancreatic cysts.^3^, ^4^ Prednisolone therapy improved the stenosis. Additionally, alcohol abstinence produced a favorable outcome.

## CONFLICT OF INTEREST

None declared.

## AUTHORSHIP

All the authors made substantial contribution to the preparation of this manuscript and approved the final version for submission. JK and AT: drafted the manuscript; AT: corresponding author; MI: clinical support; ST: careful review of the manuscript.
